# Small intestinal bacterial overgrowth and dysbiosis in children with intestinal failure: A descriptive cohort study

**DOI:** 10.1002/jpen.2808

**Published:** 2025-07-28

**Authors:** Johannes Hilberath, Andreas Busch, Ulrich Schoppmeier, Kristina Schmauder, Philipp Oberhettinger, Matthias Marschal, Christoph Slavetinsky, Ekkehard Sturm, Silke Peter, Paul D'Alvise

**Affiliations:** ^1^ Pediatric Gastroenterology and Hepatology University Children's Hospital Tübingen Tübingen Germany; ^2^ Pediatric Gastroenterology and Hepatology Children's Hospital Princess Margaret Darmstadt Darmstadt Germany; ^3^ Institute of Medical Microbiology and Hygiene University Hospital Tübingen Tübingen Germany; ^4^ Pediatric Surgery and Urology University Children's Hospital Tübingen Tübingen Germany

**Keywords:** intestinal dysbiosis, intestinal failure, next‐generation sequencing, small intestinal bacterial overgrowth

## Abstract

**Background:**

Small intestinal bacterial overgrowth (SIBO) is a clinical and diagnostic challenge in pediatric intestinal failure. This study aimed to assess SIBO and dysbiosis in children with intestinal failure and to analyze clinical characteristics as well as cultural and metagenomic sequencing results from different sampling methods.

**Methods:**

Descriptive, single‐center cohort study in intestinal failure patients with prospective collection of intraluminal aspirate, epithelial brush swab, mucosal biopsy, and small bowel stoma stool for SIBO diagnosis, defined as ≥10^3^ CFU/ml of enteric, colonic‐type bacteria, and microbiome analysis via whole‐genome sequencing. Statistical testing included receiver operating characteristic analysis, chi‐square test, and independent samples *t* test.

**Results:**

Forty‐four children with intestinal failure were analyzed (median age 58 months; female 48%; short bowel syndrome 70%). Sixty‐six percent of samples were positive for SIBO. In 93%, all three endoscopic sampling methods showed congruent results. SIBO‐positive cases were associated (*P* < 0.05) with small bowel dilatation, proton pump inhibitor use, intestinal inflammation, elevated direct bilirubin and hepatocellular enzyme levels, and a history of liver fibrosis and central venous catheter infections. Metagenomic sequencing revealed microbial dysbiosis in intestinal failure patients, with SIBO‐positive cases showing higher microbial reads, lower alpha diversity, and increased abundance of Enterobacteriaceae and enteric anaerobes.

**Conclusion:**

SIBO and dysbiosis are common in children with intestinal failure and associated with liver injury, central line–associated bloodstream infections, and intestinal inflammation. Cultural diagnosis of SIBO using mucosal biopsies or brush swabs are alternatives to small bowel aspirates. Metagenomic sequencing is feasible, and high microbial read numbers are indicative of SIBO.

## INTRODUCTION

Small intestinal bacterial overgrowth (SIBO) is a poorly understood manifestation of gut microbial dysbiosis, characterized by an excessive increase in selected bacteria within the small bowel.^1^, ^2^, ^3^ Children with intestinal failure are at risk for developing SIBO, which is associated with gastrointestinal symptoms, malabsorption, D‐lactic acidosis, infections, and liver disease.^4^, ^5^, ^6^, ^7^ As a long‐term consequence, SIBO may impair intestinal adaptation and delay parenteral nutrition weaning.^8^ The optimal threshold for diagnosing SIBO has recently changed from >10^5^ colony‐forming units (CFU)/ml to >10^3^ or ≥10^3^ CFU/ml in duodenal/jejunal fluid aspirate.^1^, ^9^, ^10^, ^11^ However, other endoscopic sampling methods, such as epithelial brush swabs or biopsies, despite being used in clinical practice,^12^ have not been compared to the gold standard for SIBO diagnosis. Furthermore, data exploring qualitative changes in the microbiome using metagenomic sequencing are limited, and there is an absence of studies comparing traditional culture‐based methods with emerging techniques, such as metagenomic sequencing, in the pediatric intestinal failure population.

Discussed disease‐specific risk factors of SIBO include an altered small bowel anatomy, ileocecal valve resection, dysmotility, stasis of gut content, parenteral nutrition, and decreased oral‐enteral feeding.^13^, ^14^, ^15^ The pathophysiology includes inflammation, villus atrophy, barrier dysfunction, bacterial translocation, and altered bile acid synthesis.^7^, ^16^ As many symptoms of SIBO are indistinguishable from those of the underlying intestinal failure disease, objective diagnosis of SIBO is recommended before considering antibiotic therapy.^17^ Notably, antibiotic treatment for SIBO itself has been linked to the development of gut dysbiosis.^13^, ^18^ For definitive SIBO diagnosis, culture‐dependent approaches with quantification of CFU of (colonic‐type) bacterial flora in a small bowel aspirate obtained by endoscopy are considered the gold standard.^2^, ^19^ Less invasive methods, such as lactose or glucose breath tests, are used for simplicity but have inferior sensitivity and specificity compared with culture.^10^, ^20^

The primary outcome of our descriptive cohort study was the assessment of SIBO, defined as ≥10^3^ CFU/ml of enteric, colonic‐type bacteria in endoscopic small bowel samples in children with intestinal failure. We further aimed to analyze the clinical and microbiological characteristics of patients with intestinal failure with and without SIBO by using and comparing cultural and metagenomic sequencing results from different endoscopic sampling methods (intraluminal aspirate, epithelial brush swab, and mucosal biopsies).

The findings of this study may have implications for diagnostic accuracy and management, as they provide a detailed analysis of the clinical and microbiological characteristics of pediatric intestinal failure patients with and without SIBO. This study could also provide new information on the evaluation of metagenomic sequencing results compared with traditional culture‐based methods for SIBO diagnosis and is the first study to compare multiple endoscopic sampling methods for SIBO diagnosis.

## METHODS

We performed a descriptive cohort study in 44 children with intestinal failure who received routine screening endoscopies at the University Children's Hospital Tübingen from 2019 to 2021. Patients with recent surgery within 6 months and those receiving intravenous antibiotics were excluded. Medical records were reviewed for data collection at time of endoscopy. The study was conducted according to the Declaration of Helsinki's ethical principles and approved by the local institutional ethics committee (281/2018BO1).

### Endoscopy

Endoscopies as routine screening procedures for children with intestinal failure were performed by an endoscopist trained in pediatric gastroenterology. No intravenous antibiotics were given prior to intervention.

**Figure 1:**
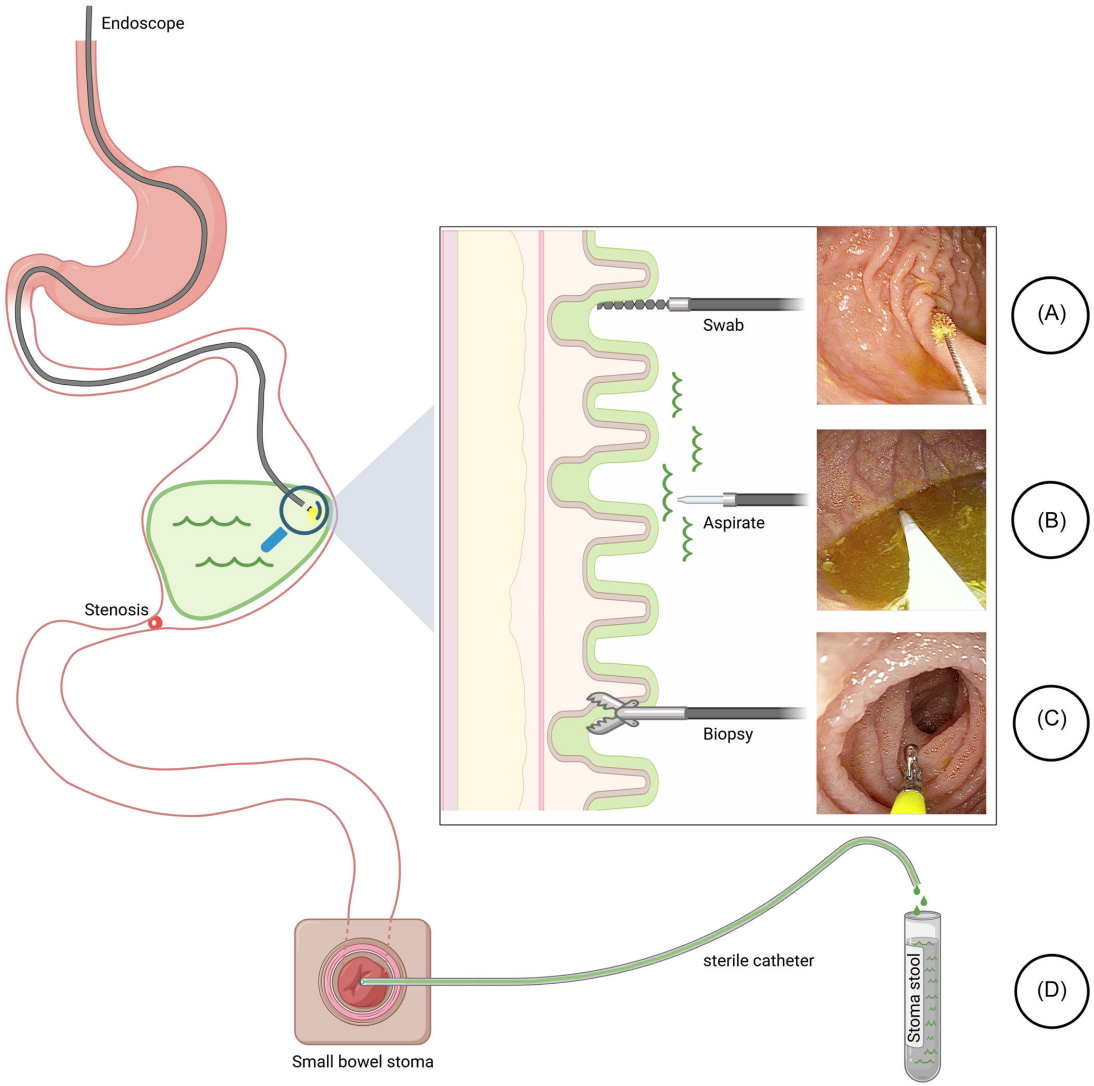
Schematic illustration of small bowel sample collection for small intestinal bacterial overgrowth diagnostic in a pediatric short bowel patient with a jejunostomy, small intestinal stenosis, proximal dilatation, and stasis: by upper jejunal endoscopy (A) epithelial swab, (B) luminal fluid aspiration, (C) mucosal biopsy, and (D) via sterile tube insertion into the distal stoma. Created with biorender.com.

Small intestinal samples for cultural and microbiome analysis were collected within the same bowel segment (Figure 1). Aspirate: 1–2 ml of intraluminal fluid were aspirated via a sterile tube through the instrument channel. Swab: epithelial swab was obtained using a cytological brush. Biopsy: mucosal biopsy was taken using biopsy forceps through the instrument channel. Additionally, in patients with a small bowel stoma, a sterile tube was inserted 4–5 cm through this stoma to collect 1–2 ml stool sample.

### Cultural microbiological analysis

Ten microliters of each small intestinal sample were plated on different selective and nonselective agar plates: Brain‐Heart‐Infusion‐Chocolate agar (47 g/L Brain‐Heart‐Agar (Oxoid) with 5% sheep blood and 10 ml BD BBL IsoVitalX (BD) added after autoclaving), MacConkey agar No.3 (Thermo Fisher), Columbia CNA agar (Biomerieux), ESBL CromID agar (Biomerieux), Cetrimide agar (45.3 g Cetrimide agar base (BD), 10 ml glycerine), and VRE ChromID agar (Biomerieux). The cultures were incubated under anoxic (Brain‐Heart‐Infusion‐Chocolate agar) or oxic atmosphere (all other media) at 37°C, and the plates were checked daily over 72 h for new colonies. Colonies with different morphologies were isolated, identified by MALDI‐TOF‐MS (Bruker MicroFlex LT‐SH).

### Definition of SIBO

Bacterial cultures were classified as positive for SIBO if ≥1 colony of enteric, colonic‐type bacteria were cultivated from the 10 µl inocula of the obtained sample, corresponding to ≥10^3^ CFU/ml. In addition, a second, higher cutoff level of ≥10^4^ CFU/ml of enteric, colonic‐type bacteria was analyzed.

### DNA extraction, whole‐genome sequencing, and Taxonomic profiling

DNA was extracted from the frozen samples, and sequenced bidirectionally using Illumina NextSeq. Microorganisms occurring at a minimal fraction of 0.1% were identified by metagenomic taxonomic profiling using a custom reference genome dataset of 170 bacterial and fungal species that inhabit the human alimentary tract. See File S1 for methodological details.

### Statistical analysis

Microsoft Excel (Microsoft Corporation) and SPSS (IBM SPSS Statistics 28, IBM) were used for data collection and analysis. Patient variables were evaluated with descriptive statistics. Receiver operating characteristic curve analysis, chi‐square test for two categorical variables, or independent samples *t* test were performed. Testing was performed with the use of a two‐sided alpha level of 0.05.

## RESULTS

Within the 2‐year study period, 92 endoscopies were performed in 62 children with chronic intestinal failure at our intestinal rehabilitation center. Six patients were excluded because of a high bleeding risk from portal hypertension. Informed consent was obtained from 44 children, and 44 endoscopies were analyzed. The main indication for gastrointestinal endoscopy was diagnostic evaluation, for example, suspected stenosis. In 10 cases, an intervention was performed, such as insertion or change of a feeding tube.

The demographics and characteristics of the study participants are presented in Table 1.

**Table 1:** Patient demographics and characteristics.

	No. of intestinal failure patients with upper endoscopy, *N*=44
Age at time of upper endoscopy, months, median [IQR]	58 [30–81]
Female, *n* (%)	21 (47.7)
*Intestinal failure etiology*	
‐ Short bowel syndrome, *n* (%)	31 (70.4)
‐ Motility disorder, *n* (%)	13 (29.6)
*Anatomy*	
‐ Residual short bowel length from the ligament of Treitz in patients with history of intestinal resection (cm, median [IQR]) *n* = 33; not known in 8 patients	50 [28–80]
‐ Presence of ileocecal valve, *n* (%)	13 (29.6)
‐ Presence of small intestinal stoma, *n* (%)	13 (29.5)
‐ Presence of small bowel dilatations, *n* (%)	25 (56.8)
‐ History of serial transverse enteroplasty procedure (STEP) in short bowel syndrome patients	5 (11.4)
*Management at time of endoscopy*	
‐ Parenteral nutrition, *n* (%)	38 (86.4)
‐ Oral‐enteral antibiotic, *n* (%)	12 (31.9)
‐ Proton pump inhibitor, *n* (%)	14 (27.3)
‐ Probiotics, *n* (%)	0 (0.0)
*Hepatopathy*	
‐ History of fibrosis (ultrasound or histopathology), *n* (%)	12 (25.5)
‐ History of steatosis (ultrasound or histopathology), *n* (%)	3 (6.4)
‐ Cholestasis (direct bilirubin ≥ 0.5 mg/dl), *n* (%)	9 (20.5)
‐ Elevated hepatocellular enzymes (AST and ALT and gGT upper normal limit), *n* (%)	9 (20.5)
*Intestinal inflammation*	
‐ Histopathological confirmed small bowel inflammation (*n* = 43, missing data in 1 case), *n* (%)	21 (48.8)
‐ Fecal calprotectin >200 µg/g stool (*n* = 25, missing laboratory data in 19 cases), *n* (%)	10 (40.0)
C‐reactive protein >1 mg/dl (*n* = 40, missing data in 4 cases), *n* (%)	5 (12.5)

Abbreviations: ALT, alanine transaminase; AST, aspartate transaminase; gGT, gamma‐glutamyltransferase.

### SIBO

In 29 cases (65.9%), at least one small bowel sample (luminal aspirate and/or mucosal biopsy and/or epithelial swab) fulfilled the diagnostic criterion of ≥10^3^ CFU/ml of enteric colonic‐type bacteria, whereas 15 cases (34.1%) were negative for all three sampling methods. Using a higher cutoff of ≥10^4^ CFU/ml of enteric colonic‐type bacteria, in 26 cases (59.1%) at least one of the three small bowel sample types were SIBO‐positive and 18 cases (40.9%) were SIBO‐negative. In two patients, data for one sample method (aspirate or swab) were missing (Table S1). No sample had to be categorized as contaminated.

In 39 of 42 cases (92.9) with a complete dataset, the culture results from all three sample types concurred when using a cutoff level of ≥10^3^ CFU/ml. Among the SIBO‐positive cases (*n* = 29), 96.4% (27 of 28), 96.4% (27 of 28), and 96.5% (28 of 29) were diagnosed with SIBO using aspirate, swab, or biopsy, respectively.

Using a higher threshold of 10^4^ CFU/ml of enteric bacteria for SIBO diagnosis (*n* = 26), 24 of 25 (96.0%), 23 of 25 (92.0%), and 24 of 26 (92.3%) patients were identified by luminal aspirate, epithelial swab, and mucosal biopsy, respectively. In 20 SIBO cases, the cultural results of all three sample types complied with ≥10^4^ CFU/ml (20 of 26, 77.0%).

In 12 children with jejunostomy or ileostomy, stoma stool cultures were analyzed: all samples were SIBO‐positive using a threshold of ≥10^3^ and ≥10^4^ CFU/ml stoma aspirate. In six of those intestinal stoma patients (50%), however, cultural SIBO diagnosis from endoscopy samples were all negative.

### Metagenome sequencing

Mucosal biopsy samples yielded the highest total sequence read numbers, whereas aspirates yielded the lowest (Table 2). Conversely, for microbial reads, aspirates showed the highest numbers and biopsies the lowest. Brush swab samples showed intermediate total and microbial read numbers but were most constant in microbial loads and yielded the highest median read number. One metagenomic sequencing dataset was missing for each sample type.

**Table 2:** Read numbers and microbial read numbers for both the different endoscopic sample types and SIBO‐positive vs SIBO‐negative cases (SIBO‐positive is defined as ≥10^3^ CFU/ml).

	Aspirate (*n* = 46), data missing in *n* = 1	Brush swab (*n* = 46), data missing in *n* = 1	Biopsy (*n* = 46), data missing in *n* = 1	Stoma aspirate (*n* = 12)
*Total reads*				
‐ All cases (*n* = 44)				
Sum	686,868,806	807,500,951	1,410,858,283	337,144,310
Median	6,795,254	16,358,584	28,204,094	24,753,554
[IQR]	[746,370, 30,522,631]	[2,160,586, 31,797,626]	[20,728,324, 39,868,172]	[13,373,832, 34,165,172]
‐ SIBO‐positive cases (*n* = 29 (66%))				
Sum	571,605,465	539,113,152	1,035,811,012	250,352,008
Median	21,702,569	13,257,190	30,174,167	22,341,404
[IQR]	[2,173,757, 33,367,107]	[2,049,017, 30,803,042]	[23,321,672, 41,617,997]	[13,373,832, 42,140,422]
‐ SIBO‐negative cases (*n* = 15 (34%))				
Sum	115,263,341	268,387,799	375,047,271	86,792,302
Median	1,087,186	17,535,436	23,642,436	25,970,591
[IQR]	[68,310, 6,795,254]	[2,210,904, 31,797,626]	[15,944,056, 39,635,082]	[12,377,130, 31,019,021]
*Microbial reads*				
‐ All cases				
Sum	293,244,790	64,118,058	28,488,669	115,011,695
Median	45,243	46,851	2785	1,661,007
[IQR]	[1,424, 11,618,274]	[3066, 481,944]	[660, 29,710]	[4123, 17,883,799]
‐ SIBO‐positive cases				
Sum	288,154,440	54,180,662	28,480,376	63,371,797
Median	7,869,705	356,322	8683	1,062,050
[IQR]	[18,298, 17,917,295]	[2946, 56,399]	[34,679, 1,748,411]	[23,722, 14,788,570]
‐ SIBO‐negative cases				
Sum	5,090,350	9,927,340	8181	51,639,898
Median	1275	477	3024	11,205,499
[IQR]	[432, 3775]	[2, 859]	[926, 13,274]	[346, 20,216,854]
*Microbial/total reads*				
‐ SIBO‐positive cases				
Overall	56,7%	5.7%	3.7%	
Median	47.5%	8.4%	0.047%	
‐ SIBO‐negative cases				
Overall	4.4%	3.7%	0.0022%	
Median	0.34%	0.05%	0.0020%	

Abbreviation: SIBO, small intestinal bacterial overgrowth.

For aspirate samples, a microbial‐per‐total‐reads ratio of ≥10.6% showed a sensitivity of 85.7% and a specificity of 80% for predicting a SIBO‐positive case (area under the curve [AUC]: 0.857; data missing in *n* = 1).

For biopsy samples, a microbial‐per‐total‐reads ratio of ≥0.005% showed a sensitivity of 82.1% and specificity of 73.3% for predicting a SIBO‐positive case (AUC: 0.852; data missing in *n* = 1).

For brush swab samples, a microbial‐per‐total‐reads ratio of ≥0.19% showed a sensitivity of 82.1% and specificity of 73.3% for predicting a SIBO‐positive case (AUC: 0.776; data missing in *n* = 1).

**Figure 2:**
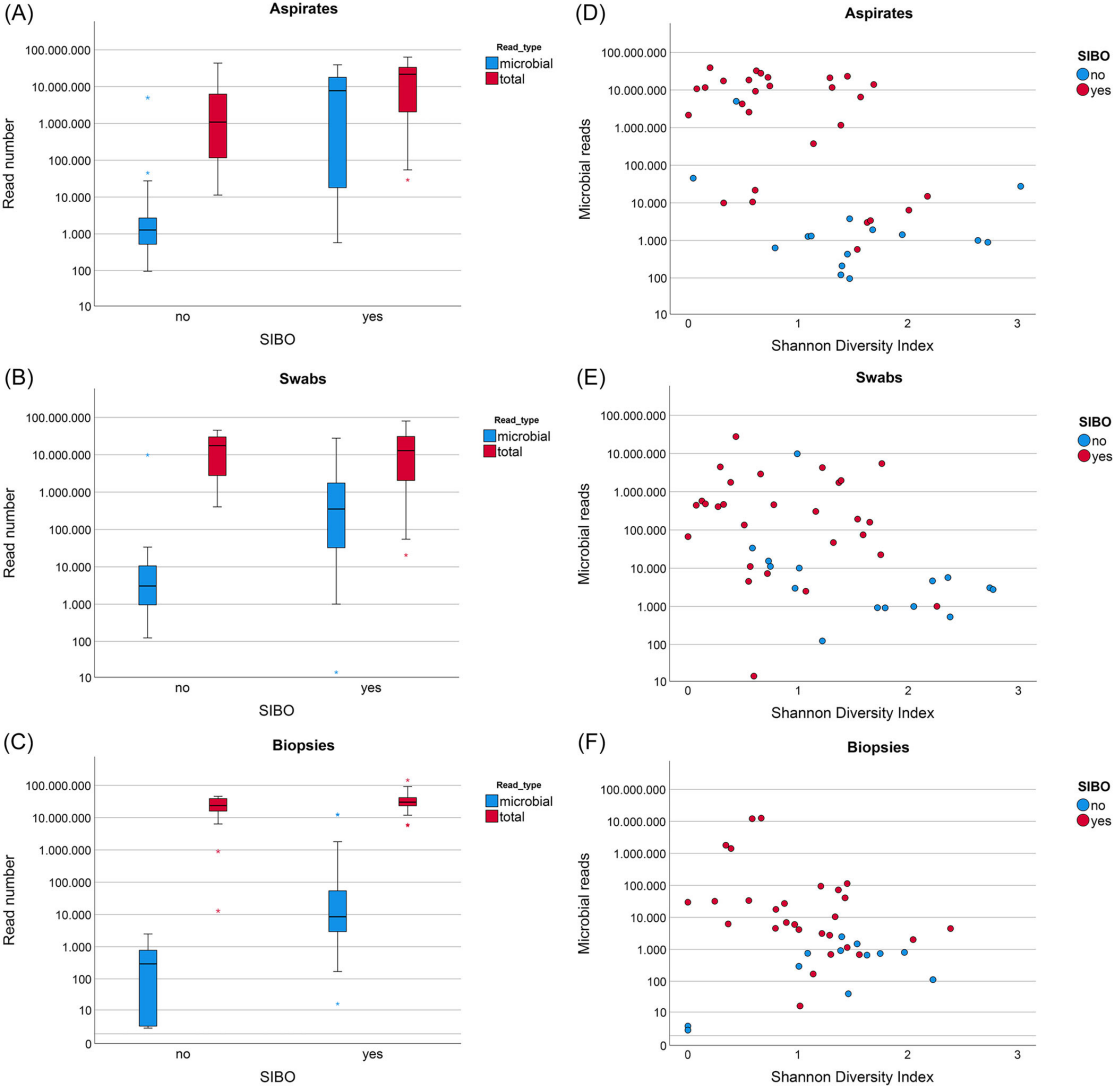
(A–C) Box‐and‐whisker diagrams comparing total and microbial read numbers in small intestinal bacterial overgrowth (SIBO) patients and non‐SIBO patients from luminal aspirate samples (A), brush swab samples (B), and biopsies (C). Solid lines within boxes indicate median values, box borders show 25th to 75th percentiles, error bars 5th and 95th percentiles, and stars represent outliers. (D–F) Scatter diagrams of microbial reads (*y*‐axis) vs Shannon diversity indices (x‐axis) for SIBO‐positive and negative cases in luminal aspirates (A), brush swabs (B), and biopsies (C). Dense microbial communities dominated by one or few species of Enterobacteriaceae in the samples from SIBO‐positive patients result in higher microbial read numbers and lower alpha diversity.

**Figure 3:**
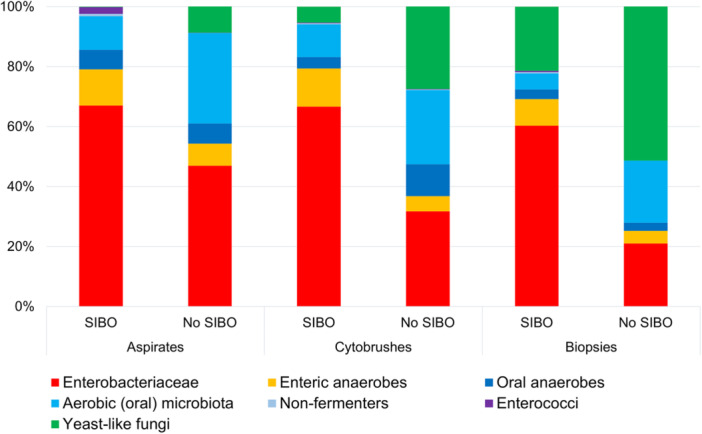
Microbial community profiles of small intestinal bacterial overgrowth (SIBO) patients and non SIBO patients. In SIBO‐positive patients, microbial communities were skewed toward Enterobacteriaceae (red) and enteric anaerobes (orange).

Compared with SIBO‐negative cases, most SIBO‐positive cases showed high microbial read numbers in biopsy, aspirate, and brush swab samples (Figure 2) and showed reduced alpha diversity (Shannon diversity index), indicating highly skewed microbial communities with one or very few dominant microbes (Figure 2D–E). Sequencing of aspirate and swab samples from SIBO‐positive patients yielded millions of microbial reads, accounting for median fractions of 48% (aspirates) and 8% (swabs) of total reads (Table 2). In SIBO‐negative patients, median fractions of microbial reads were 0.3% and 0.06% for aspirates and swabs, respectively. The same pattern was observed for the biopsy samples that, as expected, contain mostly human reads; for SIBO‐positive samples, the median percentage of microbial reads in the biopsies was 0.05%, compared with 0.002% for SIBO‐negative patients. Microbial communities of SIBO‐positive patients are usually dominated by one or few species of enteric microbes, typically *Enterobacteriaceae* like *E coli* or *Klebsiella spp*, resulting in lower Shannon diversity indices compared with non‐SIBO samples (Figure 2D–E). Microbial community profiles of SIBO patients were skewed toward *Enterobacteriaceae* (red) (Figure 3) and enteric anaerobes (orange), for example, *Clostridium spp* or *Bacteroides spp*. Non‐SIBO microbial profiles show higher relative amounts of oral or skin microbiota (blue), such as staphylococci or streptococci, and oral anaerobic bacteria like *Prevotella spp* or *Fusobacterium spp*. Culture‐negative samples usually yielded low numbers of microbial reads from oral or skin‐dwelling bacteria or yeasts (green), likely representing DNA from dead cells.

### Clinical features in children with and without SIBO

There were no differences in the etiology of intestinal failure between patients with and without SIBO based on endoscopically obtained samples (Table 3); however, children with SIBO showed more frequent small bowel dilatations. The use of proton pump inhibitor (*P* = 0.02) and parenteral nutrition (*P* = 0.07) was more common in the SIBO group. Elevated direct bilirubin levels were noted only in children with SIBO (*P* = 0.02). Increased hepatocellular enzyme levels (AST and ALT and gGT) at the time of endoscopy and a history of liver fibrosis were more prevalent in SIBO‐positive cases (*P* = 0.02 and *P* = 0.003, respectively). Children with SIBO had a history of central venous catheter infections more often (*P* = 0.01). Although fecal calprotectin did not differ between groups, histopathologically confirmed intestinal small bowel inflammation was more prevalent in SIBO‐positive cases (*P* = 0.03).

**Table 3:** Comparison of clinical features in patients with intestinal failure with and without SIBO based either on cultural samples obtained by endoscopy or based on stoma stool samples.

	SIBO [endoscopy samples] (*n* = 29)	No SIBO [endoscopy samples] (*n* = 15)	*p* value*	SIBO [stoma stool samples] (*n* = 12)	No SIBO [stoma stool and endoscopy samples] (*n* = 9)	*p* value*
*Intestinal failure etiology*						
Short bowel syndrome, *n* (%)	21 (72.4)	10 (66.7)	0.69	4 (33.3)	7 (77.8)	0.04
Motility disorder, *n* (%)	8 (27.6)	5 (33.3)	0.69	8 (66.7)	2 (22.2)	0.04
*Anatomy at time of endoscopy*						
‐ Presence of ileocecal valve, *n* (%)	6 (20.7)	6 (40.0)	0.17	0 (0.0)	6 (66.7)	<0.001
‐ Presence of small intestinal stoma, *n* (%)	7 (24.1)	6 (40.0)	0.27	12 (100)	0 (0.0)	<0.001
‐ Presence of small bowel dilatations, *n* (%)	20 (69.0)	5 (33.3)	0.02	4 (33.3)	3 (33.3)	1.0
‐ History of serial transverse enteroplasty procedure (STEP) in short bowel syndrome patients (*n* = 21)	5 (23.8)	0/11 (0.0)	0.14	0 (0.0)	0 (0.0)	N/A
*Management at time of endoscopy, n* (%)						
‐ Parenteral nutrition, *n* (%)	27 (93.1)	11 (73.3)	0.07	11 (91.7)	6 (66.7)	0.15
‐ Oral‐enteral antibiotic, *n* (%)	11 (37.9)	3 (20.0)	0.12	4 (34.4)	0 (0.0)	0.05
‐ Proton pump inhibitor, *n* (%)	12 (41.4)	1 (6.7)	0.02	2 (16.7)	1 (11.1)	0.72
‐ Probiotics, *n* (%)	0 (0.0)	0 (0.0)	N/A	0 (0.0)	0 (0.0)	N/A
‐ Tube feeding, *n* (%)	9 (31.0)	2 (13.3)	0.20	3 (25.0)	2 (22.2)	0.88
‐ Oral feeding, *n* (%)	27 (93.1)	15 (100.0)	0.30	11 (91.7)	9 (100)	0.38
*Hepatopathy*, *n* (%)						
‐ History of fibrosis	12 (41.4)	0 (0.0)	0.003	2 (16.7)	0 (0.0)	0.20
‐ History of steatosis	3 (10.3)	0 (0.0)	0.20	1 (8.3)	0 (0.0)	0.34
‐ Cholestasis (direct bilirubin ≥ 0.5 mg/dl)	9 (31.0)	0 (0.0)	0.02	3 (25.0)	0 (0.0)	0.11
‐ Hepatocellular enzymes (AST and ALT and gGT) upper normal limit	9 (31.0)	0 (0.0)	0.02	7 (58.3)	2 (22.2)	0.10
*Intestinal inflammation*, *n* (%)						
‐ Histopathological confirmed small bowel inflammation	17 (60.7); data missing in 1	4 (26.7)	0.03	7 (63.6); data missing in 1	2 (22.2)	0.06
‐ Fecal calprotectin >200 µg/g stool	9 (50.0); data missing in 11	1 (14.3); data missing in 8	0.10	0 (0.0); data missing in 9	1 (33.3); data missing in 6	0.27
C‐reactive protein >1 mg/dl	4 (15.4); data missing in 3	2 (14.3); data missing in 1	0.93	2 (22.2); data missing in 3	2 (22.2)	1.0
History of CLABSI in patients with PN	14/27 (51.9)	1/12 (8.3)	0.01	4/11 (36.4)	0/7 (0.0)	0.07

*Note*: SIBO diagnosis based on positive culture results (≥10^3^ CFU/ml).

Abbreviations: CFU, colony‐forming units; CLABSI, central line–associated bloodstream infection; N/A, not applicable; PN, parenteral nutrition; SIBO, small intestinal bacterial overgrowth.

* Chi‐square test for two categorical variables.

When comparing SIBO‐positive cases based solely on small intestinal stoma stool samples with SIBO‐negative cases, there were no differences in management, hepatopathy, intestinal inflammation, laboratory values, and central line–associated bloodstream infection events.

## DISCUSSION

SIBO is common in children with intestinal failure; however, as there are no clinical consensus criteria to define or treat SIBO, it remains a relevant clinical and diagnostic challenge, with limited data in this unique group of patients. This single‐center study is one of the five largest analyses of SIBO in pediatric patients with intestinal failure,^10^ describing its frequency, risk factors, and clinical features. To the best of our knowledge, this is the first study to compare different cultural sampling methods to the gold standard of duodenal aspirate for SIBO diagnosis. In parallel with the cultural SIBO diagnosis, samples were obtained and analyzed for metagenome sequencing.

Based on the gold standard of duodenal aspirates obtained by endoscopy and a threshold of ≥10^3^ CFU/ml of enteric bacteria, 27 of 43 (63%) samples in our cohort were diagnosed with SIBO. Swab brushes and mucosal biopsies diagnosed SIBO in 27 of 43 (63%) and 28 of 44 (64%) of cases, respectively. Including the SIBO‐negative cases, the results of the three sample methods obtained by endoscopy showed congruent results in 93%. Therefore, swab brushes and biopsies can be considered alternatives for the cultural diagnosis or exclusion of SIBO, which has not been previously investigated. The importance of this finding is highlighted by the fact that, in clinical practice, intestinal rehabilitation providers already use different methods for SIBO diagnosis, including small bowel brushings and biopsies.^12^

The frequency of SIBO in our cohort is in line with a recent systematic review that reported a SIBO prevalence of 57.5% in patients with intestinal failure.^10^ However, as the included endoscopic studies used a cutoff of >10^5^ CFU/ml in duodenal fluid, a direct comparison is not possible. Therefore, it is noteworthy that an optimal threshold of ≥10^3^ CFU/ml duodenal aspirate as used in our study was identified for the diagnosis of SIBO.^9^

Various risk factors for the development of SIBO have been identified, including altered intestinal anatomy including resection of the ileocecal valve, parenteral nutrition dependency, intestinal dysmotility, and proton pump inhibitor–induced hypochlorhydria.^4^, ^21^ In that regard, the presence of small bowel dilatation was more prevalent in SIBO‐positive cases in our cohort. It is known that stasis of intestinal contents may lead to SIBO.^8^ Thus, it can be assumed that motility disorders, such as pediatric intestinal pseudo‐obstruction, predispose patients to SIBO.^22^ However, we could not show a difference in etiology (short bowel syndrome vs motility disorders) between patients with and without SIBO. This could be explained by the fact that 75% of our patients with motility disorders have a small intestinal stoma for prevention of stasis. On the other hand, all five patients with a history of a bowel‐lengthening procedure (serial transverse enteroplasty procedure [STEP]) were SIBO‐positive. Four of them (80.0%) demonstrated small bowel dilatation, indicating a possible role of STEP as a risk factor for redilatation and (consecutive) SIBO development. As gastric acid plays an important role in preventing bacteria from colonizing the proximal GI tract, medication‐induced hypochloridria by proton pump inhibitor is a known risk factor for SIBO.^2^ We also found a more frequent use of antacids in the SIBO‐positive group of our cohort.

Metagenomic sequencing was feasible for all three endoscopic sample methods. Mucosal biopsies yielded the highest total read numbers, due to a higher load of human reads than in brush swabs and aspirates. Microbial read numbers varied greatly, from very few (<10) in culture‐negative biopsy samples to millions in SIBO‐positive aspirate samples. Generally, microbial read numbers were considerably higher in the SIBO‐positive cases than in the SIBO‐negative cases. Therefore, a high number of microbial reads strongly indicated SIBO within this study, but this metric alone cannot compare sequencing results between studies or serve as diagnostic criterion. Methodological differences in sampling and sequencing can lead to vast variation in read numbers, so the microbial read number should be regarded in relation to the total read number as proxy for sequencing depth.

This study is, to our knowledge, the first to correlate metagenomic sequencing with culture‐based SIBO diagnostics in pediatric intestinal failure patients. We suggest that predicting a culture‐based SIBO diagnosis is possible via the ratio of microbial read number to total read number, that is, the percentage of microbial reads, with cutoff values of ≥10.6% for aspirates (sensitivity 86%, specificity 80%), ≥0.2% for swabs (sensitivity 82%, specificity 73%), and ≥0.005% for biopsies (sensitivity 82%, specificity 73%).

Metagenomic sequencing revealed microbial dysbiosis in children with intestinal failure. In SIBO‐positive cases, there were higher microbial read numbers, reduced alpha diversity, communities predominantly composed of Enterobacteriaceae, and a greater abundance of enteric anaerobes. The results suggest that there were both oral and colon‐specific anaerobic bacteria present in some of the samples and that these bacteria are usually not recovered by routine cultural diagnostics, which is, in principle, a limitation of the cultural method. However, this did not compromise the diagnostic sensitivity of the cultural method, since the colon‐specific anaerobes were always found together with considerably higher numbers of *Enterobacteriaceae*, which function as the primary indicator of SIBO. For this reason, we conclude that disproportionately elaborate measures to ensure quantitative survival of strictly anaerobic bacteria are not necessary.

Decreased bacterial diversity with an abundance of Enterobacteriaceae has been published in nine children with short bowel syndrome via 16S rRNA gene sequencing of fecal samples.^23^ Interestingly, in that study, dysbiosis was associated with prolonged parenteral nutrition dependence. In our larger cohort of children using endoscopic small bowel samples analyzed by metagenomic sequencing, we also found a trend toward greater parenteral nutrition dependency in SIBO‐positive cases.

SIBO has been linked to intestinal inflammation, barrier dysfunction, and bacterial translocation.^10^, ^24^ We had the unique opportunity to support this notion by our study, in which biopsies for histopathology were obtained within the same small bowel segment: In SIBO‐positive cases, histopathologically confirmed inflammation was found more frequently than in SIBO‐negative samples. In addition, in our cohort, a history of central line–associated infection was also more likely in SIBO‐positive cases. It remains speculative whether these central line–associated bloodstream infection events were a consequence of SIBO or if they caused intestinal dysbiosis following anti‐infective medication. However, published literature supports the former thesis: Cole et al demonstrated in infants with short bowel syndrome that SIBO increases the risk of bloodstream infections.^6^ On a microbiome level, this is further supported by the study of Talathi et al, which showed changes in the gut microbiome from prior to the occurrence of a central line–associated bloodstream infection event.^18^

Dysbiosis and SIBO have also been linked to intestinal failure–associated liver disease (IFALD).^18^ Altered gut microbiome composition and intestinal barrier function appear to contribute to hepatopathy development and progression in patients with intestinal failure.^7^ Our findings support this association in children: SIBO was more likely in patients with a history of hepatic fibrosis. In addition, in patients with SIBO, the prevalence of elevated direct bilirubin and hepatocellular enzymes was higher than in SIBO‐negative cases. These laboratory results, obtained at the time of endoscopy, highlight the possible role of SIBO‐mediated liver injury. In their review, Mihajlovic et al discuss anaerobic bacteria–derived hepatotoxins as a contributing factor in IFALD pathogenesis.^7^

In our cohort, the frequency of prophylactic antibiotics did not differ between SIBO‐negative and positive cases, highlighting that enteral antibiotics do not appear to eradicate SIBO. Instead, Talathi et al showed that scheduled antibiotics lead to a reduced microbial diversity and increased abundance of *Proteobacteria* in fecal samples.^18^ Although our findings suggest caution in the use of antibiotics, it is important to contrast this with a recent study showing symptom improvement after antibiotic therapy in children with intestinal failure and SIBO.^5^

All stool samples from small bowel stoma were SIBO‐positive, even using a higher cultural threshold of ≥10^4^ colonic‐type CFU/ml. However, the clinical significance remains unclear, as, unlike SIBO diagnosis from duodenal samples, there were no significant differences in the studied clinical outcome parameters. Therefore, a SIBO‐positive result based on stoma stool samples should be interpreted cautiously.

Our study had several limitations. It lacked a (healthy) control group. Comparisons of children with and without intestinal failure with regard to microbial alterations have been published before.^6^, ^23^, ^25^ The strength of our study lies in comparing SIBO‐positive and SIBO‐negative cases by investigating different endoscopic sample methods for culture and metagenomic sequencing. Clinical data on patient history relied on chart reviews, including intestinal anatomy and central line–associated infections. However, most data were prospectively collected, including laboratory data, intestinal histopathological results, and microbiological diagnostics. Another limitation is the small study group, although it is one of the largest published cohorts regarding SIBO and dysbiosis in children with intestinal failure.

## CONCLUSION

In conclusion, this study confirms SIBO and dysbiosis as frequent findings in children with intestinal failure and demonstrates their possible clinical relevance in IFALD, central line–associated bloodstream infection, and intestinal inflammation. It shows that cultural diagnosis or exclusion of SIBO with mucosal biopsies and brush swabs obtained by endoscopy are alternatives to the gold standard of small bowel aspirates. In contrast, the clinical relevance of SIBO diagnosis by culture‐positive small‐intestinal stoma samples is questionable. Similarly, using enteral antibiotics to eradicate SIBO in children with intestinal failure is not supported by our findings. Metagenomic sequencing highlights dysbiosis in SIBO cases by high microbial read numbers and reduced bacterial diversity.

## AUTHOR CONTRIBUTIONS

**Johannes Hilberath**: Conceptualization; investigation; writing—original draft; visualization; methodology; writing—review and editing; project administration; formal analysis. **Andreas Busch**: Conceptualization; supervision; methodology. **Ulrich Schoppmeier**: Formal analysis; methodology; conceptualization. **Kristina Schmauder**, **Philipp Oberhettinger**: Writing—review and editing; investigation. **Matthias Marschal**: Conceptualization; methodology; investigation. **Christoph Slavetinsky**: Writing—review and editing; methodology. **Ekkehard Sturm**: Supervision; writing—review and editing. **Silke Peter**: Supervision; conceptualization; project administration; writing—review and editing; resources. **Paul D'Alvise**: Writing—review and editing; visualization; validation; investigation; methodology; formal analysis; data curation.

## CONFLICT OF INTEREST STATEMENT

None declared.

## Supporting information

JPEN SIBO in pIF Table S1 Rev 1 clean version 20250603.

Supporting File for review 20250410.
