# Supplementary material for: Small intestinal bacterial overgrowth and dysbiosis in children with intestinal failure: A descriptive cohort study
Source: JPEN J Parenter Enteral Nutr. 2025 Jul 28;49(8):964–74. doi: 10.1002/jpen.2808 (PMC12581465; doi:10.1002/jpen.2808)
Supplement: Supplementary file 1 — JPEN SIBO in pIF Table S1 Rev 1 clean version 20250603. [file JPEN-49-964-s001.docx]

**Table S1. Overview of microbial culture results for the three endoscopic sample methods (luminal aspirate, epithelial swab, and mucosal biopsy) and small bowel stoma aspirate.** +, SIBO-positive (green). -, SIBO-negative (red). *CFU, colony-forming units.* -, data missing. *N/A, not applicable (grey). SIBO, small intestinal bacterial overgrowth.*

| **#** | **Aspirate_culture** | | **Swab_culture** | | **Biospy_culture** | | **Small bowel stoma aspirate** | |
| --- | --- | --- | --- | --- | --- | --- | --- | --- |
|  | *≥10^3^ CFU/mL* | *≥10^4^ CFU/mL* | *≥10^3^ CFU/mL* | *≥10^4^ CFU/mL* | *≥10^3^ CFU/mL* | *≥10^4^ CFU/mL* | *≥10^3^ CFU/mL* | *≥10^4^ CFU/mL* |
| 1 | + | + | + | + | + | + | N/A | N/A |
| 2 | - | - | + | - | + | - | + | + |
| 3 | + | + | + | + | + | + | + | + |
| 4 | + | + | + | + | + | + | N/A | N/A |
| 5 | + | + | *-* | *-* | + | + | + | + |
| 6 | + | + | + | + | + | + | N/A | N/A |
| 7 | + | + | + | + | + | + | + | + |
| 8 | + | + | + | + | + | + | N/A | N/A |
| 9 | *-* | *-* | + | + | + | + | N/A | N/A |
| 10 | - | - | - | - | - | - | + | + |
| 11 | + | + | + | + | + | + | N/A | N/A |
| 12 | + | + | + | + | + | + | N/A | N/A |
| 13 | - | - | - | - | - | - | + | + |
| 14 | - | - | - | - | - | - | N/A | N/A |
| 15 | - | - | - | - | - | - | N/A | N/A |
| 16 | + | + | + | + | + | - | N/A | N/A |
| 17 | + | + | + | + | + | + | N/A | N/A |
| 18 | + | + | + | + | + | + | N/A | N/A |
| 19 | + | + | + | - | + | + | N/A | N/A |
| 20 | - | - | - | - | - | - | N/A | N/A |
| 21 | + | + | + | + | + | + | N/A | N/A |
| 22 | + | + | + | + | + | + | + | + |
| 23 | + | + | + | + | + | + | N/A | N/A |
| 24 | - | - | - | - | - | - | + | + |
| 25 | - | - | - | - | - | - | + | + |
| 26 | - | - | - | - | - | - | N/A | N/A |
| 27 | - | - | - | - | - | - | N/A | N/A |
| 28 | + | + | + | + | + | - | + | + |
| 29 | + | - | - | - | + | - | N/A | N/A |
| 30 | + | + | + | + | + | + | N/A | N/A |
| 31 | - | - | - | - | - | - | + | + |
| 32 | + | - | + | - | - | - | N/A | N/A |
| 33 | - | - | - | - | - | - | N/A | N/A |
| 34 | + | + | + | + | + | + | N/A | N/A |
| 35 | + | + | + | + | + | + | N/A | N/A |
| 36 | + | + | + | + | + | + | N/A | N/A |
| 37 | + | + | + | + | + | + | N/A | N/A |
| 38 | - | - | - | - | - | - | N/A | N/A |
| 39 | + | + | + | + | + | + | N/A | N/A |
| 40 | - | - | - | - | - | - | N/A | N/A |
| 41 | - | - | - | - | - | - | N/A | N/A |
| 42 | + | + | + | + | + | + | N/A | N/A |
| 43 | - | - | - | - | - | - | + | + |
| 44 | + | - | + | - | + | + | N/A | N/A |
