# Supplementary material for: Small intestinal bacterial overgrowth and dysbiosis in children with intestinal failure: A descriptive cohort study
Source: JPEN J Parenter Enteral Nutr. 2025 Jul 28;49(8):964–74. doi: 10.1002/jpen.2808 (PMC12581465; doi:10.1002/jpen.2808)
Supplement: Supplementary file 2 — Supporting File for review 20250410. [file JPEN-49-964-s002.pdf]

Supplemental file for review

Detailed description of methods

Cultural microbiological analysis

10µl of each small intestinal sample were plated on different selective and non-selective agar plates: Brain-Heart-Infusion-Chocolate agar (47g/L Brain-Heart-Agar (Oxoid) with 5% sheep blood and 10 mL BD BBL IsoVitalX (BD) added after autoclaving), MacConkey agar No.3 (Thermo Fisher), Columbia CNA agar (Biomerieux), ESBL CromID agar (Biomerieux), Cetrimide agar (45,3g Cetrimide agar base (BD), 10ml glycerine), and VRE ChromID agar (Biomerieux). The cultures were incubated under anoxic (Brain-Heart-Infusion-Chocolate agar) or oxic atmosphere (all other media) at 37℃, and the plates were checked daily over 72h for new colonies. Colonies with different morphologies were isolated, identified by MALDI-TOF-MS (Bruker MicroFlex LT-SH), and tested for antibiotic susceptibility according to EUCAST guidelines (eucast.org), using automated antibiotic sensitivity testing (Vitek 2, Biomerieux) and agar-diffusion assays.

DNA extraction and whole-genome sequencing

The frozen (-80°C) samples were thawed, and DNA was extracted using the DNeasy UltraClean Microbial Kit (Qiagen, Hilden, Germany). The genomic DNA was sheared (Covaris M220, Woburn, USA) to obtain 550-bp fragments, and libraries were prepared using the TruSeqNano DNA LT Kit (Illumina, San Diego, USA) according to the manufacturer’s standard protocol. The prepared barcoded libraries were quantified on the Invitrogen Qubit 4 fluorometer (Thermo Fisher Scientific, Germany) and analyzed on the QIAxcel Advanced capillary electrophoresis instrument (Qiagen, Hilden, Germany). All libraries were sequenced bidirectionally on an Illumina NextSeq instrument with 2 x 150bp reads using the NextSeq 500/550 High Output Kit v2.5 (300 cycles) (Illumina, San Diego,USA). Quality control was performed using FastQC version 0.11.9 (Babraham Bioinformatics).

Taxonomic profiling

The high-quality reads were subjected to taxonomic profiling on the CLC Genomic Workbench v.22.0 using a custom-built microbial database. A primary taxonomic profiling analysis using the large default database of 5268 microbial reference genomes was run on the reads of all samples. The resulting preliminary taxonomic profiles were used to identify the bacterial species occurring in the samples and to select 170 reference genomes of species that occur in at least one of the samples at a relative proportion of 0.1% of the microbial reads and are known to inhabit the human alimentary tract. Thereby, odd taxa that were identified in the preliminary taxonomic analysis, but likely represent database bias artifacts, were eliminated; e.g. *Shigella flexneri* in any sample containing *E. coli, Yersinia pestis, Edwardsiella tarda, Chania multitudinisentens, Thermogemmatispora carboxidivorans*. Shannon indices for diversity were calculated for each sample.
